# Supplementary material for: Longitudinal examination of young married women’s fertility and family planning intentions and how they relate to subsequent family planning use in Bihar and Uttar Pradesh India
Source: BMJ Open. 2022 Jul 21;12(7):e064487. doi: 10.1136/bmjopen-2022-064487 (PMC9310155; doi:10.1136/bmjopen-2022-064487)
Supplement: Author's manuscript [file bmjopen-2022-064487.draft_revisions.pdf]

BMJ Open

BMJ Open is committed to open peer review. As part of this commitment we make the peer review history of every article we publish publicly available.

When an article is published we post the peer reviewers’ comments and the authors’ responses online. We also post the versions of the paper that were used during peer review. These are the versions that the peer review comments apply to.

The versions of the paper that follow are the versions that were submitted during the peer review process. They are not the versions of record or the final published versions. They should not be cited or distributed as the published version of this manuscript.

BMJ Open is an open access journal and the full, final, typeset and author-corrected version of record of the manuscript is available on our site with no access controls, subscription charges or pay-per-view fees (http://bmjopen.bmj.com).

If you have any questions on BMJ Open’s open peer review process please email info.bmjopen@bmj.com

BMJ Open

What does 'unmet need' have to do with it? Young married women's fertility and family planning needs in Bihar and Uttar Pradesh India

Journal:	*BMJ Open*
Manuscript ID	bmjopen-2022-064487
Article Type:	Original research
Date Submitted by the Author:	08-May-2022

Complete List of Authors:	Speizer, Ilene; University of North Carolina at Chapel Hill Gillings School of Global Public Health, Maternal and Child Health<br>Zavier, A.J.; Population Council India<br>Calhoun, Lisa; University of North Carolina at Chapel Hill, Carolina Population Center,<br>Nanda, Priya; Bill and Melinda Gates Foundation India<br>Saggurti, Niranjan; Population Council<br>Hainsworth, Gwyn; Bill & Melinda Gates Foundation

Keywords:	PUBLIC HEALTH, STATISTICS & RESEARCH METHODS, REPRODUCTIVE MEDICINE

SCHOLARONE™ Manuscripts

I, the Submitting Author has the right to grant and does grant on behalf of all authors of the Work (as defined in the below author licence), an exclusive licence and/or a non-exclusive licence for contributions from authors who are: i) UK Crown employees; ii) where BMJ has agreed a CC-BY licence shall apply, and/or iii) in accordance with the terms applicable for US Federal Government officers or employees acting as part of their official duties; on a worldwide, perpetual, irrevocable, royalty-free basis to BMJ Publishing Group Ltd ("BMJ") its licensees and where the relevant Journal is co-owned by BMJ to the co-owners of the Journal, to publish the Work in this journal and any other BMJ products and to exploit all rights, as set out in our licence.

The Submitting Author accepts and understands that any supply made under these terms is made by BMJ to the Submitting Author unless you are acting as an employee on behalf of your employer or a postgraduate student of an affiliated institution which is paying any applicable article publishing charge ("APC") for Open Access articles. Where the Submitting Author wishes to make the Work available on an Open Access basis (and intends to pay the relevant APC), the terms of reuse of such Open Access shall be governed by a Creative Commons licence – details of these licences and which Creative Commons licence will apply to this Work are set out in our licence referred to above.

Other than as permitted in any relevant BMJ Author's Self Archiving Policies, I confirm this Work has not been accepted for publication elsewhere, is not being considered for publication elsewhere and does not duplicate material already published. I confirm all authors consent to publication of this Work and authorise the granting of this licence.

For peer review only

What does 'unmet need' have to do with it? Young married women's fertility and family planning needs in Bihar and Uttar Pradesh India

Ilene S. Speizer,[1,2*] A. J. Francis Zavier,[3] Lisa M. Calhoun,[1] Priya Nanda,[4] Niranjan Saggurti,[3] Gwyn Hainsworth[4]

*Corresponding author: Ilene S. Speizer, Department of Maternal and Child Health, Gillings School of Global Public Health, University of North Carolina at Chapel Hill, Chapel Hill, NC, USA. ilene_speizer@unc.edu

Abstract

Objectives: Among young married women from Bihar and Uttar Pradesh India, the site of this study, unmet need is high (about 49%); however, family planning (FP) use is only 9%. Alternative measures are needed to identify those young women who are the most likely to adopt a family planning method.

Design: We use three-year longitudinal data from married women ages 15-19 in 2015-16 (Wave 1) who are not using contraception to examine factors associated with any use of FP in 2018-19 (Wave 2).

Results: Multivariate analyses demonstrate that young women who want to delay childbearing three or more years or who do not want any(more) children at Wave 1 are more likely to use contraception at Wave 2. Additionally, intention to use FP in the next 12 months at Wave 1 is significantly associated with FP use at Wave 2 whereas unmet need at Wave 1 is not significantly related to subsequent use. A combined measure of fertility desires and intention to use FP in the next 12 months demonstrates the importance of both fertility desires and FP use intentions on subsequent use. Having any children and being pregnant at Wave 1 are both related to Wave 2 use.

Conclusions: To support young, married women's attainment of their fertility desires, it is important to reach them prior to a first pregnancy with nuanced messages addressing their intentions and ability to use FP in the future. Further, programs targeting women at antenatal and postpartum visits are likely to be important for young women to help support them to delay or limit future childbearing for the health and well-being of themselves and their children.

For peer review only - http://bmjopen.bmj.com/site/about/guidelines.xhtml

Strengths and limitations of this study

- Using longitudinal data permits identifying meaningful measures of fertility desires and family planning use intentions for young, married women.
- The results from this study can be used to better inform program targeting of the women with the greatest contraceptive needs.
- Findings demonstrate that recently married young women need information and services (at the time of marriage, prior to a birth, or at antenatal, or postpartum care) to affect their agency and intentions to use contraception in the future.
- The study is limited because a key measure, intention to use family planning in the next 12 months, does not correspond to the three-year follow-up period.
- Some women may have adopted a method between the two survey waves, and this was not captured with these data; this is a limitation of this analysis.

For peer review only

For peer review only - http://bmjopen.bmj.com/site/about/guidelines.xhtml

What does 'unmet need' have to do with it? Young married women's fertility and family planning needs in Bihar and Uttar Pradesh India

Introduction

Early and closely spaced pregnancies can have long term negative health and well-being outcomes for young mothers and their children.[1] In places where the age at marriage is low and gender norms dictate immediate childbearing post marriage, understanding factors associated with young women's (and couples') use of FP to delay and space births is helpful for understanding how to develop programs that lead to improved individual, maternal, and child health outcomes.

At the aggregate level, unmet need for family planning (FP) is a policy variable, demonstrating hypothetical gaps between fertility desires and contraceptive use. Unmet need is defined as the population of fecund, sexually active women of reproductive age who report a desire to avoid childbearing 2+ years or not have any(more) children and are not using contraception.[2] Recent studies that examine the association between unmet need, fertility desires, and subsequent fertility and FP behaviors demonstrate that there is a weak link between unmet need and outcomes.[3-5] In a study using longitudinal data from Uganda, Sarnak and colleagues[3] demonstrate that among non-contracepting women in 2014, those with an unmet need are slower to adopt a contraceptive method over the four-year follow-up period than those without an unmet need. The authors also find that intentions to use contraception in the future are significantly related to contraceptive adoption.[3] Importantly, using a combined measure of unmet need and intentions to use contraception in the future, women who intend to use, whether or not they have an unmet need, have a shorter time to adoption in the follow-up period. Further, in a study among urban women from six cities in Uttar Pradesh, India, Speizer and colleagues[5] demonstrate that in a two-year follow-up period, women who did not want any(more) children were significantly less likely to have a birth than all other women; this effect was bigger among

For peer review only - http://bmjopen.bmj.com/site/about/guidelines.xhtml

those using contraception at baseline. However, no difference in birth experience is found between women who wanted to space and women who wanted a pregnancy soon, no matter their baseline contraceptive use. These results are consistent with an earlier study from India by Roy and colleagues[6] that found that over a six-year follow-up period, many women did not adhere to their fertility preferences nor to their FP use intentions.

Recently, colleagues have proposed the importance of considering intention to use contraception in addition to unmet need to better assess actual gaps in FP use.[7,8] Moreau and colleagues[7]propose a measure called current status unmet demand that removes currently pregnant and recently postpartum women and incorporates women's intentions to use contraception in the future. In aggregate estimations of women of all ages across 46 countries, adjusted unmet need is lower (13.8%) than the standard unmet need (16.4%) and only 6.7% of women have an unmet need and intend to use and 7.2% have an unmet need but do not intend to use in the future.[7] Similarly, using data from India, Panda and colleagues[8] identify those women of all ages who have an unmet need and intend to use FP in the next 12 months and demonstrate that this group is important for identifying women most in need of FP.

Prior studies focus on all women of reproductive ages (ages 15-49). Less is known about the fertility desires and FP use intentions of adolescents (ages 15-19); what is known among young women typically comes from cross-sectional studies.[9-13] Young women are of particular interest since they are at the beginning of their reproductive lives and some may want to delay a first birth or space births to pursue their aspirations (e.g., stay in school, work, get to know their husbands) or for better maternal and child health outcomes. It is important to examine young women in the India context where at the time of the 2019-2021 National Family Health Survey (NFHS) 16% of women ages 20-24 in Uttar Pradesh[14] and 41% of their counterparts from Bihar[15] were married before the legal minimum age of marriage of 18; these young married women have often already begun childbearing and thus may have underserved FP needs.

For peer review only

This study is unique because it uses longitudinal data collected among adolescents from Bihar and Uttar Pradesh, India. Longitudinal data permit the examination of how fertility desires and FP use intentions among married adolescents evolve over the adolescent and young adult years with the other changes underway in these young peoples' lives. The study also permits examination of various measures of fertility desires, unmet need, and FP intentions and their programmatic utility for identifying young women with FP needs.

Methods

Study sample

Data for this study come from two states in northern India – Bihar and Uttar Pradesh. Both states have large populations, are among the poorest in the country, and are predominately rural.[16] This study uses secondary data collected as part of a study led by the Population Council in India called "Understanding the Lives of Adolescent and Young Adults," also called the UDAYA study. In 2015-2016, Wave 1 data were collected among a representative sample of unmarried girls and boys (ages 10-19) and married girls ages 15-19. More details on the study design can be found elsewhere[17,18] and on the project website (www.projectudaya.in). Three years later (between 2018-2019), Wave 2 follow-up data were collected with the sample of participants who were interviewed in Wave 1.

This analysis focuses on the sample of women ages 15-19 who were married with Gauna performed (i.e., the marriage is consummated) at Wave 1 who were followed in Wave 2 and had information on the fertility and FP variables of focus in this analysis. The full sample of young women surveyed in Wave 1 was 4,893 (see Table 1). A total of 4,000 of these women were re-interviewed in Wave 2 (81.7%) while 893 women (18.3%) were not found at Wave 2. Table 1 presents the characteristics of the full sample, the analysis sample (n=4,000) and the sample lost-to-follow-up. The women who were not interviewed at Wave 2 tended to be from Uttar Pradesh (p<0.001), have no births at Wave 1 (p=0.10), and be from

For peer review only - http://bmjopen.bmj.com/site/about/guidelines.xhtml

urban areas ($p<0.001$). In the analysis of which young married women were using FP at Wave 2, the sample is limited to married adolescents who were non-users at Wave 1 (n=3,616) and had full information on the explanatory variables (n=3,614).

Outcome variable

The key outcome variable is use of any contraceptive method at Wave 2. Women who at Wave 2 reported using any modern method (pill, IUD, injectable, implant, condom, diaphragm, foam/jelly, female or male sterilization, female condom, or LAM) or any traditional method (rhythm method and withdrawal) were coded 1. All others were coded zero, including those who were pregnant at Wave 2 and those who reported never using a method at Wave 2. Among the full sample at Wave 2 (n=4,000), 17% were using any method and 10% were using a modern method. The analyses presented here focus on any method use; however, models were also run with the outcome of modern method use and the results were similar.

For peer review only

For peer review only - http://bmjopen.bmj.com/site/about/guidelines.xhtml

Table 1. Descriptive information on sample of married[a] participants at Wave 1 and the longitudinal sample for analysis

	Full sample at Wave 1[a] (n=4,893)	Wave 2 follow-up sample (n=4,000; 81.7%)	No follow-up sample (n=893; 18.3%)	p-value*
**State (%)**				
Uttar Pradesh	60.4	58.1	71.0	<0.001
Bihar	39.6	42.0	29.0	
**Age in years (%)**				
15	1.9	1.8	2.5	NS
16	6.7	6.9	5.8	
17	15.6	15.6	15.6	
18	32.5	32.6	31.8	
19	43.3	43.1	44.3	
**Parity (%)**				
None	61.0	59.9	66.0	0.10
1	31.8	32.3	29.2	
2+	7.2	7.8	4.8	
**Education (%)**				
None	24.8	24.2	27.6	NS
1-4 years	5.9	5.7	7.0	
5-7 years	16.7	16.7	17.0	
8-9 years	26.4	27.0	23.8	
10-11 years	12.7	12.7	12.6	
12+ years	13.4	13.7	12.2	
**Wealth (%)**				
Lowest	15.2	14.8	16.9	NS
Low	20.8	21.3	18.8	
Middle	23.5	23.3	24.6	
High	23.7	24.0	22.5	
Highest	16.7	16.6	17.3	
**Religion (%)**				
Hindu	82.3	83.0	79.0	NS
Muslim	17.5	16.8	20.7	
Others	0.2	0.2	0.3	
**Caste (%)**				
Scheduled Castes/Tribes	30.0	29.7	31.1	NS
Other Backward Castes	57.5	58.1	54.8	
General	12.5	12.2	14.1	
**Residence (%)**				
Urban	10.8	9.7	16.1	<0.001
Rural	89.2	90.3	83.9	

All values weighted using Wave 1 weights. *F-test comparing analysis sample to those lost to follow-up.
[a]Sample that is married with Gauna performed.

For peer review only - http://bmjopen.bmj.com/site/about/guidelines.xhtml

For peer review only

Key independent variables

To understand fertility desires and FP use intentions of young married women, we examine whether variables from Wave 1 are associated with use of FP at Wave 2, among non-users at Wave 1. First, we examine Wave 1 desire for a future pregnancy and the timing of that desire (see Table 2 for these variables). This is categorized as: wants a pregnancy before 2 years (soon), wants in 2 years, wants in 3 years, wants in 4+ years, undecided, and wants no more children. Second, we include the standard measure of unmet need (no vs. yes), calculated based on questions about fertility desires, FP use, and intentionality of a recent/last pregnancy as traditionally defined.[2] We also include two questions on intention to use FP – one about intention to use in the next 12 months, and the other about intention to ever use in the future; both of these are coded as no, yes, or don't know. For the intention to use FP questions, women who had ever used a method and were current non-users were not asked this question (n=75); for this analysis, these women were coded as intends to use based on their prior experience. In models that dropped the women with missing information, the results are like those presented. In this Indian context where the norm is to have 2-3 children and then get sterilized, it is not surprising that most young women intend to ever use in the future (81% at Wave 1); therefore, for this analysis, we focus on intention to use in the next 12 months, even though the follow-up period is 3 years.

For peer review only - http://bmjopen.bmj.com/site/about/guidelines.xhtml

Table 2. Fertility and family planning variables at Wave 1 and Wave 2 of analysis sample of women ages 15-19 and married at Wave 1

	Wave 1<br>%	Wave 2<br>%
**Desire for a pregnancy**		
Wants within 2 years	31.3	20.8
Wants in 2 years	18.4	12.5
Wants in 3 years	15.0	8.9
Wants in 4+ years	12.8	8.0
Undecided	10.2	8.3
Wants no more	12.3	41.4
**Currently using any method**	n=3,994*	
No	91.0	82.8
Yes	9.0	17.2
**Unmet need**		n=3,965**
No	51.4	54.3
Yes	48.6	45.7
**Currently pregnant**		
No	78.8	83.2
Yes	21.2	16.8
**Intention to use FP ever in the future**	n=3,998*	
Yes	81.3	89.8
No	14.4	9.1
Don't know	4.3	1.2
**Intention to use FP in next 12 months**	n=3,998*	
Yes	48.2	62.2
No	46.1	36.9
Don't know	5.7	0.9
**Experience birth or pregnancy between Waves (includes currently pregnant at Wave 2)**	NA	
No		13.32
Yes		86.7
**Intentionality of most recent birth or pregnancy experienced**	NA	n=3,461
Wanted then		65.6
Wanted later		31.2
Wanted no more		3.2

Unweighted number of observations shown is 4,000 unless shown otherwise. * Two women missing information on this measure at Wave 1. **35 women missing relevant information for Wave 2 unmet need measure. Wave 1 and Wave 2 values use Wave 2 weights that adjust for loss to follow-up.

For peer review only - http://bmjopen.bmj.com/site/about/guidelines.xhtml

In response to recommendations by Moreau and colleagues[7] and Panda and colleagues[8] we create measures that jointly capture fertility desires and intentions to use contraception in the future. First, we create a combined measure based on fertility desires and intentions to use FP in the future with six distinct categories (see Table 3). Second, we create a combined measure based on unmet need and intention to use FP in the future; this variable has four categories that can be seen in Table 3. All women who reported that they did not know if they intend to use in the next 12 months were grouped with "no intention to use."

For peer review only

For peer review only - http://bmjopen.bmj.com/site/about/guidelines.xhtml

Table 3. Fertility and family planning variables of non-users with full information at Wave 1 and the correlation with Wave 2 family planning use

Wave 1 characteristic	Wave 1 non-users %	Percent using FP at Wave 2	
**Desire for a pregnancy**			
Wants within 2 years	33.0	7.9	p<0.001
Wants in 2 years	18.6	12.5	
Wants in 3 years	14.3	16.5	
Wants in 4+ years	11.8	16.6	
Undecided	11.0	13.9	
Wants no more	11.4	26.2	
**Unmet need**			
No	46.6	11.6	p=0.006
Yes	53.4	15.6	
**Intention to use FP in next 12 months**			
Yes	43.0	17.6	p<0.001
No	50.8	9.9	
Don't know	6.3	18.5	
**Fertility desire/FP use intention†**			
Wants now/no intention	32.2	9.1	p=0.001
Wants now/intention to use	11.7	10.1	
Wants later/no intention	20.2	11.0	
Wants later/intention to use	24.5	18.0	
Wants no more/no intention	4.6	22.3	
Wants no more/intention to use	6.8	28.8	
**Unmet need/FP use intention**			
No unmet need/no intention	30.7	9.8	p=0.001
No unmet need/intention to use	15.9	15.3	
Unmet need/no intention to use	26.3	12.1	
Unmet need/intention to use	27.1	19.0	

This table focuses on the non-users of any method at Wave 1 (n=3,614). All percentages weighted using Wave 2 weights. Significance testing determines if family planning use differs by the various Wave 1 fertility desire and intention to use categories. † Wants now includes those wanting in two years or undecided; wants later is those who want to delay 2+ years; no intention includes those who are undecided on future use.

We also descriptively examine if women experienced a birth between Wave 1 and Wave 2 (or are currently pregnant at Wave 2) and the intentionality of the recent birth or current pregnancy as

For peer review only - http://bmjopen.bmj.com/site/about/guidelines.xhtml

reported at Wave 2. This information is useful for better understanding the utility of assessing fertility desires among young, married women in Bihar and Uttar Pradesh and the fluidity of these desires.

Demographic variables

Because fertility desires and contraceptive behaviors are closely tied to a woman's current pregnancy status, we adjust for whether the woman is currently pregnant at Wave 1 (no vs. yes); this avoids dropping currently pregnant women from the analyses. We also adjust for Wave 1 state (Bihar vs. Uttar Pradesh), caste (Scheduled Caste, Scheduled Tribe, Other Backward Caste, General), age in years, parity (no children, one child, two or more), education level (none, primary, 8-9 years of education, 10+ years of education), religion (Hindu vs. all others), wealth group (lowest, low, medium, high, highest), and place of residence (urban vs. rural). The coding and distribution of these variables is shown in Table 1.

Analysis

Univariate and bivariate analyses are used to examine the associations between fertility desires and FP use intentions at Wave 1 and Wave 2 contraceptive use. All descriptive statistics that examine the characteristics of the Wave 1 sample, including examination of loss to follow-up, use Wave 1 weights. All analyses that focus on the longitudinal sample use Wave 2 weights to adjust for loss to follow-up. Multivariate logistic regression analyses are used to inform which young non-users at Wave 1 are the most likely to use any FP method at Wave 2, adjusting for Wave 1 demographic characteristics. Recognizing that some of the young women are pregnant at Wave 1 and may not need FP at that time; instead of dropping them as typically done, we have controlled for this Wave 1 pregnancy to see how this relates to use at Wave 2. All multivariate analyses are unweighted but adjust for sample clustering. This study uses secondary de-identified data and was deemed exempt from ethical review by the University of North Carolina Institutional Review Board.

For peer review only - http://bmjopen.bmj.com/site/about/guidelines.xhtml

For peer review only

Patient and public involvement: Patients and/or the public were not involved in the design or conduct or reporting or dissemination plans of this research.

Results

Table 1 shows that the sample of married adolescents is predominately ages 18 and 19 at Wave 1 (mean age is 18.0 years); this is consistent with the legal age of marriage for women in India. By Wave 2, the cohort is ages 17-23 with a mean age of 20.8 years (not shown). More than half of the sample has not had any children and one third has one child; the remaining women (about 8%) have had two or more children by the time of the Wave 1 survey. About a quarter of the sample has no education while about half has 8+ years of education. The majority of the sample is Hindu, and most participants live in rural areas. Finally, more than half of the sample is Other Backward Castes and a quarter are Scheduled Castes/Tribes.

Table 2 presents a comparison of the fertility and FP variables for the longitudinal sample; all results use the Wave 2 weights. At Wave 1, a third of women wants a child soon (within 2 years) and another third wants a child in 2 or 3 years. Nearly 10% of women are undecided about future childbearing. Thirteen percent of women report that they want to wait 4+ years before having a(nother) child and 12% say they do not want (any)more children. By Wave 2, a greater percentage of women want no more children as many have given birth between waves (87% experience a pregnancy or birth). Only 9% of this young sample is using any method of contraception at Wave 1. That said, for the unmet need measure that is based on fertility desires, contraceptive use, and intentionality of last/recent pregnancies we find that 48.6% of these young women have an unmet need for FP. At Wave 1, 21% of the sample is currently pregnant; these women may have a future need for FP, following the delivery of their baby.

For peer review only - http://bmjopen.bmj.com/site/about/guidelines.xhtml

Also included in Table 2 is the intention to use FP (ever or in the next 12 months). In this India context where sterilization is common, it is not surprising that more than four-fifths intend to use FP ever in the future. Yet only 48% of these young women intend to use FP in the next 12 months.

Table 2 includes the same measures at Wave 2 to examine aggregate changes in the three-year follow-up period. As mentioned above, 87% of women had a birth between waves or are currently pregnant at the time of the Wave 2 survey. Most of the births/pregnancies were wanted then (66%) and one third were wanted "later," that is the woman wanted to wait 2+ years before the birth. This high birth experience between waves is likely to affect the other fertility and FP measures assessed at Wave 2. Notably, while contraceptive use goes up between surveys (9% to 17%), it does not increase as much as the desire to avoid pregnancy (12% to 41%). Unmet need at Wave 2 remains high (46%). Finally, intentions to use FP ever in the future and in the next 12 months also increased by Wave 2.

Table 3 focuses on the non-users of contraception at Wave 1 and presents their fertility and FP variables as well as how this corresponds to FP use at Wave 2. Based on Wave 1 fertility desires, those young women who want no more children were more likely to be using by Wave 2 (27%) than all other fertility desire groups; this is followed by those who want to wait 3+ years before the next birth (16.5%). Based on unmet need at Wave 1, a significantly greater percentage of those with an unmet need at Wave 1 are using at Wave 2 (16%) compared to those without an unmet need at Wave 1 (12%); however, this difference is smaller than would be expected given the importance assigned to this global indicator. As expected, a significantly greater percentage of young women who report at Wave 1 that they intend to use FP in the next 12 months (18%) are using at Wave 2 compared to those who say they do not intend to use in the next 12 months (10%); those who are unsure if they will use are also more likely to use (19%).

Table 3 also includes a combined fertility desire and intention to use variable. Here, we find that women who want no more children at Wave 1 are the most likely to be using at Wave 2. In addition,

For peer review only - http://bmjopen.bmj.com/site/about/guidelines.xhtml

among those who do not want any more children, those who intend to use are more likely to be using (29%) than those who do not intend to use (22%). Intentions also differentiate those who want to delay a subsequent birth such that those who want to delay and intend to use are more likely to be using at Wave 2 (18%) compared to those who want to delay but do not intend to use (11%). Table 3 includes the combined unmet need and intention to use measure. Here we find that 19% of young women with an unmet need and intention to use in the next 12 months are using at Wave 2 compared to 10% among those with no unmet need and no intention to use and 12% among those with an unmet need and no intention to use; interestingly, 15% of those with no unmet need but an intention to use were using at Wave 2.

Table 4 presents the multivariable logistic regression odds ratios (OR) and 95% confidence intervals (CI) for the analysis of Wave 2 use of any contraception among non-users at Wave 1, including Wave 1 fertility desires and FP intentions and adjusting for Wave 1 demographics. Model 1 shows that women who at Wave 1 want to wait 4+ years (OR: 1.68; 95% CI: 1.19-2.37) and those women who do not want any(more) children (OR: 2.27; 95% CI: 1.58-3.25) were significantly more likely to be using a method at Wave 2 than women who want a child within 2 years. In addition, women who want to wait 3 years were also significantly more likely to be using at Wave 2. No difference is found in Wave 2 use between women who were undecided about future childbearing and women who want a child in two years as compared to women who at Wave 1 want a child soon. Also included in this model is if the woman was pregnant at Wave 1; those women who were pregnant were significantly more likely to be using at Wave 2 than those who were not pregnant. Further, women who have no children at Wave 1 were significantly less likely to be using at Wave 2 and those with 2+ children were significantly more likely to be using at Wave 2 compared to women with one child. The other Wave 1 demographics (age, education, caste, religion, place of residence, and wealth quintile) were in the expected directions and

For peer review only

not shown here. Findings for Wave 1 pregnancy, parity and the demographics are consistent across all models shown (contact first author for these results).

For peer review only

For peer review only - http://bmjopen.bmj.com/site/about/guidelines.xhtml

Table 4. Examination of use of any contraception at Wave 2 based on Wave 1 characteristics and Wave 1 fertility desires/FP intentions (among non-users at Wave 1, n=3,614)

	**Model 1**	**Model 2**	**Model 3**	**Model 4**	**Model 5**
	OR (95% CI)	OR (95% CI)	OR (95% CI)	OR (95% CI)	OR (95% CI)
Fertility Desires					
Wants within 2 years (ref)	1.0				
Wants in 2 years	1.17 (0.83-1.66)				
Wants in 3 years	1.54 (1.08-2.20)*				
Wants in 4+ years	1.68 (1.19-2.37)**				
Undecided	1.33 (0.90-1.97)				
Wants no more	2.27 (1.58-3.25)***				
Intention to use FP in 12 months					
No (ref)		1.0			
Yes		1.33 (1.08-1.64)**			
Don't know		1.32 (0.87-2.01)			
Unmet need					
No (ref)			1.0		
Yes			1.16 (0.96-1.40)		
Fertility desire/FP use intention					
Wants now/no intention (ref)				1.0	
Wants now/intention to use				0.98 (0.68-1.42)	
Wants later/no intention				1.08 (0.82-1.42)	
Wants later/intention to use				1.42 (1.08-2.64)*	
Wants no more/no intention				1.69 (1.08-2.64)*	
Wants no more/intention to use				2.20 (1.55-3.13)***	
Unmet need/FP use intention					
No unmet/no intention (ref)					1.0
No unmet/intention					1.36 (1.01-1.83)*
Unmet need/no intention					1.19 (0.92-1.55)
Unmet need/intention					1.43 (1.09-1.86)**

Note: All models also control for Wave 1 pregnancy status, Wave 1 parity, state, caste, age, education, religion, place of residence, and wealth; +p≤0.10; *p≤0.05; ***p≤0.001. Multivariate models are unweighted but adjust for sample clustering.

For peer review only - http://bmjopen.bmj.com/site/about/guidelines.xhtml

Model 2 presents the same model with intention to use contraception in the next 12 months as the key variable. Adjusting for demographic factors, those women who at Wave 1 intend to use FP soon are significantly more likely to be using at Wave 2 (OR: 1.33; 95% CI: 1.08-1.64) than those who do not intend to use FP in the next year.

Model 3 includes the unmet need measure at Wave 1. There is no difference in use at Wave 2 between those with no unmet need and those with an unmet need at Wave 1.

Model 4 includes the combined fertility desire and intention to use FP variable. Those women who want to avoid childbearing at Wave 1 are the most likely to use, no matter their Wave 1 intention to use FP. In addition, those women who want to delay childbearing 2+ years and who report an intention to use in the next 12 months were significantly more likely to be using at Wave 2 than their counterparts who want a child soon and did not intend to use. That said, no difference in Wave 2 use is found between those who want to delay childbearing 2+ years but do not intend to use and those who want a child soon and do not intend to use.

Finally, Model 5 includes the combined unmet need and intention to use FP measure. Those women who have an unmet need and intend to use FP in the next 12 months have higher odds of using (OR: 1.43; 95% CI: 1.09-1.86) than those women with no unmet need and no intention to use. Further, women who at Wave 1 have no unmet need and an intention to use are also more likely to be using at Wave 2 (OR: 1.36; 95% CI: 1.01-1.83) than those with no unmet need and no intention to use.

Finally, Table 5 provides additional context on how fertility desires among the young women play out in actual behaviors. Among women who at Wave 1 want a child in two years or are undecided about future childbearing, over 90% became pregnant or had a birth between waves. Among those who wanted to wait 4+ years or did not want any(more) children, a significantly lower percentage (82% and 81%, respectively) became pregnant or had a birth between waves. Notably, among those who want a child soon (within 2 years), only 84% became pregnant; this might be related to sub-fecundity among

For peer review only - http://bmjopen.bmj.com/site/about/guidelines.xhtml

these women who may have been trying to get pregnant for a while. Also shown in Table 5 is the intentionality of experienced pregnancies. While the results are significantly different across groups (p<0.001), it is notable that most births/pregnancies experienced are considered wanted then (58%-73%) or wanted later (26%-38%); only a small percentage of pregnancies were considered unwanted and this was predominately among those who did not want to become pregnant at Wave 1 (10%). This table indicates post-hoc rationalization of pregnancies among these young women who are early in their childbearing years.

Table 5. Pregnancy experience between Waves and intentionality of pregnancy among those who experienced a pregnancy/birth at Wave 2

		Intentionality of birth/pregnancy at Wave 2 (%) (n=3,461)			
**Wave 1: Desire for a pregnancy**	**% had birth or pregnancy between waves**	**Wanted then**	**Wanted later**	**Did not want**	**Total % (n)**
Wants within 2 years	84.5	72.9	26.3	0.9	100 (n=996)
Wants in 2 years	92.2	65.3	32.1	2.7	100 (n=659)
Wants in 3 years	88.7	61.2	37.1	1.7	100 (n=542)
Wants in 4+ years	82.0	57.6	38.4	4.1	100 (n=424)
Undecided	93.4	66.5	28.6	4.9	100 (n=432)
Wants no more	81.0	60.1	30.0	9.9	100 (n=408)
	p<0.001		p<0.001		

Uses Wave 2 weights. For percent that had a birth, n=4,000 women married at Wave 1 that also have Wave 2 data.

Discussion

This paper compares a variety of fertility desire and FP intention measures and their association with subsequent contraceptive adoption among married adolescents in Bihar and Uttar Pradesh, India; two states with poor health outcomes for mothers and babies. Using longitudinal data collected as part of the UDAYA study, we focus on women ages 15-19 who were married and not using contraception at the

For peer review only - http://bmjopen.bmj.com/site/about/guidelines.xhtml

initial interview in 2015-16 (Wave 1) and examine transitions in their contraceptive behaviors by 2018-19 (Wave 2) based on their Wave 1 fertility desires and FP use intentions.

The descriptive findings from this study demonstrate that over the follow-up period, 87% of the sample had a birth between waves or was pregnant at Wave 2; this suggests the importance of early childbearing among young, married couples from Bihar and Uttar Pradesh. Further, pregnancy/birth experience between waves is associated with Wave 1 fertility desires. For example, women who reported a desire for no more children and women who wanted to avoid a child 4+ years were the least likely to get pregnant; however more than 80% of these women experienced a birth/pregnancy between waves. Sixty percent of pregnancies/births experienced in the follow-up period to those who said they did not want any(more) children were considered “wanted then” and another 30% were “wanted later;” only 10% reported these pregnancies/births as not wanted. Prior studies from India have demonstrated that fertility desires of women of all ages (not just adolescents as included here) are fluid and there is post-hoc rationalization of pregnancies/births experienced.[5,6,18] With the data available, it is not possible to know if the women changed their fertility desires between waves, if their partner or someone else influenced their pregnancy experience, if they lacked access to contraception to meet their needs, if they did not have the agency to act on their fertility desires, or if there are other reasons for the inconsistent pregnancy/birth experience in the follow-up period.

From the data available, it appears that there are FP needs in this sample of young women. At Wave 1, only 9% of women are using a contraceptive method; however, unmet need for FP is high (49%). Unmet need in the UDAYA sample is higher than in the 2015/2016 India NFHS where 22.5% of women ages 15-19 in union from Uttar Pradesh[20] and 29.6% of their counterparts from Bihar[21] had an unmet need for FP. Further, while about 40% of these young women report an intention to use contraception in the next 12 months, FP use only increases by about 8 percentage points between Wave 1 and Wave 2. Whether the gap between intention and actual use reflects fluid adoption and discontinuation of

For peer review only - http://bmjopen.bmj.com/site/about/guidelines.xhtml

methods in the follow-up period, a lack of demand for methods (e.g., concerns about side effects), or barriers to access to methods when women are in need, is difficult to assess with the data available. A recent paper by Senderowicz and Maloney[22] proposes a strategy to divide unmet need into two categories: supply side versus demand side unmet need; this would help to better assess barriers to use. Further, Panda and colleagues[8] propose that incorporating intention to use FP into unmet need measures, as done here, may help with identifying those women with the most need in India.

In our multivariate analyses, unmet need at Wave 1 is not related to Wave 2 contraceptive use. This is not surprising given that unmet need is meant to be used at a population level and is an investigator determined and not woman-determined assessment of need.[2,22,23] That said, fertility desires matter such that those women who report a desire to delay 3+ years and those women who want to avoid childbearing are more likely to be using at Wave 2 than those women who want a child soon. Intention to use FP in the next 12 months is also related to Wave 2 contraceptive use. The combined fertility desire/intentions variable shows that those women who want to delay or avoid childbearing and intend to use at Wave 1 are the most likely to be using at Wave 2. Further, we find both descriptively and in multivariate analyses that those young women who have an unmet need and intention to use FP are more likely to use as are women who have no unmet need but an intention to use; these results are similar to those by Sarnak and colleagues[3] for all women of reproductive ages in Uganda and support the recommendations of Panda and colleagues.[8]

This study is not without limitations. An important limitation is that the intention to use FP in the next 12 months variable does not correspond to the three-year follow-up period. Given the high intention to use ever in the future, the 12-month variable seemed more relevant for this analysis. Second, we are not able to examine the timing of adoption in the follow-up period given that calendar data were not available. Further, it was not possible to examine if women adopted and discontinued in

For peer review only - http://bmjopen.bmj.com/site/about/guidelines.xhtml

the follow-up period to meet their fertility desires. Future studies that include calendar data on timing of adoption/use of FP as part of longitudinal data are important to overcome these limitations.

Conclusion

Regarding future measurement of fertility desires and FP intentions among young women, unmet need on its own is not a meaningful indicator; it is difficult to calculate, not meant to be used at an individual level, and not useful among young women who are at the beginning of their reproductive careers. That said, understanding young women's fertility desires with a more detailed measure (i.e., more categories than just want soon, want later, and does not want as typically used) is informative for understanding their "plans" for future childbearing, even if they may not be able to follow through on these fertility desires, as shown here. Further, examining young women's intentions to use a method in the next 12 months is a meaningful and simple indicator that can be used individually or in combination with fertility desires to help inform who is most likely to adopt a method in the future; this is useful for program targeting.

Our analyses also demonstrate that women who were currently pregnant at Wave 1 and/or had already had one or two pregnancies by the time of the Wave 1 survey were significantly more likely to be using at Wave 2 than all others. This reflects the norm in India that young, married couples should have a birth (or two) prior to using FP.[24,25] FP programs seeking to reach young, married women in India should promote FP messages in the antenatal and postpartum periods to ensure that there are no missed opportunities for provision of FP services.[26] While these women may not have a "current need," they do have an imminent need and may be candidates for immediate postpartum contraception, including long-acting reversible contraception .

The women who were the least likely to adopt a FP method by Wave 2 reported at Wave 1 that they wanted a child soon, did not have any children, and/or did not intend to use in the future. Programs

For peer review only - http://bmjopen.bmj.com/site/about/guidelines.xhtml

targeting these young women who may want to delay briefly (1-2 years) or longer (3+ years), should focus on demand side programming to help build young women's agency to act on their family, fertility and life course desires. This could happen through outreach or mass media programs that have messages about the benefits of FP for couple relationship strengthening, school and work attainment, as well as for the health of the mother and the child. These types of demand creation programs may address gaps in knowledge and access, increase intention to use in the future, and increase the agency of women to seek a method when or if they so desire. This may require training frontline workers on having conversations about FP with young, recently married women, their male partners and/or other gatekeepers such as mothers-in-law as well as training them to provide contraceptive methods or refer those who want to use a method they do not offer. Over time, increased engagement with young women may lead to delayed first births and better spacing between young women's births and lead to better maternal and child health and well-being.

For peer review only - http://bmjopen.bmj.com/site/about/guidelines.xhtml

References

1. Norton M, Chandra-Mouli V, Lane C. Interventions for preventing unintended, rapid repeat pregnancy among adolescents : A review of the evidence and lessons from high-quality evaluations. Global Health Science and Practice. 2017; 5(4): 547-570.
2. Bradley SEK and Casterline JB. Understanding unmet need: History, theory, and measurement. Studies in Family Planning. 2014; 45(2): 123-150.
3. Sarnak D, Tsui A, Makumbi F, Kibira SPS, Ahmed S. The predictive utility of unmet need on time to contraceptive adoption: A panel study of non-contracepting Ugandan women. Contraception. 2020; 2. https://doi.org/10.1016/j.conx.2020.100022.
4. Sarnak DO, Tsui A, Makumbi F, Kibira SPS, Ahmed S. A panel study of fertility preferences and contraceptive dynamics in the presence of competing pregnancy risks in Uganda. Demography. 2021; 58(1): 295-320. doi:10.1215/00703370-8937285.
5. Speizer IS, Calhoun L, Hoke T, Sengupta R. Measurement of unmet need for family planning: Longitudinal analysis of the impact of fertility desires on subsequent childbearing behaviors among urban women from Uttar Pradesh India. Contraception. 2013; 88(4):553–560. http://dx.doi.org.libproxy.lib.unc.edu/10.1016/j.contraception.2013.04.006.
6. Roy TK, Ram F, Nangla P, Saha U, Khan N. Can women's childbearing and contraceptive intentions predict contraceptive demand? Findings from a longitudinal study in central India. International Family Planning Perspectives. 2003; 29(1): 25-31.
7. Moreau C, Shankar M, Helleringer S, Becker S. Measuring unmet need for contraception as a point prevalence. BMJ Global Health 2019;4:e001581. doi:10.1136/bmjgh-2019-001581.
8. Panda BK, Ram F, Singh G, Saggurti N. Intention to use contraception among women in unmet need and not in unmet need in India. Demography India 2020; 41(2): 22-34.

For peer review only - http://bmjopen.bmj.com/site/about/guidelines.xhtml

9. Chandra-Mouli V, McCarraher DR, Phillips SJ, Williamson NE, Hainsworth G. Contraception for adolescents in low and middle income countries: Needs, barriers, and access. Reproductive Health. 2014; 11(1). https://doi.org/10.1186/1742-4755-11-1.
10. Ahinkorah BO. Predictors of unmet need for contraception among adolescent girls and young women in selected high fertility countries in sub-Saharan Africa: A multilevel mixed effects analysis. PLoS ONE. 2020; 15(8): e0236352. https://doi.org/10.1371/journal.pone.0236352.
11. Behrman JA, Wright KQ, Grant MJ, Soler-Hampejsek E. Trends in modern contraceptive use among young adult women in sub-Saharan Africa 1990 to 2014. Studies in Family Planning. 2018; 49(4):319–44. https://doi.org/10.1111/sifp.12075 PMID: 30431643.
12. Makola L, Mlangeni L, Mabaso M, Chibi B, Sokhela Z, Silimfe Z, et al. Predictors of contraceptive use among adolescent girls and young women (AGYW) aged 15 to 24 years in South Africa: results from the 2012 national population-based household survey. BMC Women's Health. 2019; 19(1):158. https://doi.org/10.1186/s12905-019-0861-8 PMID: 31830982.
13. Sidze EM, Lardoux S, Speizer IS, Faye CM, Mutua MM, Badji F. Young women access and use of contraception: The role of providers' restrictions in urban Senegal. International Perspectives on Sexual and Reproductive Health. 2014; 40(4):176. https://doi.org/10.1363/4017614 PMID: 25565345.
14. International Institute for Population Sciences (IIPS) and ICF. 2021. National Family Health Survey (NFHS)-5, State and District Factsheets, Uttar Pradesh. Mumbai: IIPS.
15. International Institute for Population Sciences (IIPS) and ICF. 2020. National Family Health Survey (NFHS)-5, State and District Factsheets, Bihar. Mumbai: IIPS.
16. International Institute for Population Sciences (IIPS) and ICF. 2021. National Family Health Survey (NFHS-5), India 2019-2020. Mumbai: IIPS.

For peer review only - http://bmjopen.bmj.com/site/about/guidelines.xhtml

17. Santhya, K. G., R. Acharya, N. Pandey et al. 2017. *Understanding the lives of adolescents and young adults (UDAYA) in Uttar Pradesh, India.* New Delhi: Population Council.

18. Santhya, K. G., R. Acharya, N. Pandey et al. 2017. Understanding the lives of adolescents and young adults (UDAYA) in Bihar, India. New Delhi: Population Council.

19. Koenig MA, Acharya K, Singh S, Roy TK. Do current measurement approaches underestimate levels of unwanted childbearing? Evidence from rural India. Population Studies. 2006;60(3):243–56.

20. International Institute for Population Sciences (IIPS) and ICF. 2017a. National Family Health Survey (NFHS-4), India, 2015-16: Uttar Pradesh. Mumbai: IIPS.

21. International Institute for Population Sciences (IIPS) and ICF. 2017b. National Family Health Survey (NFHS-4), India, 2015-16: Bihar. Mumbai: IIPS.

22. Senderowicz L, Maloney N. Suppl-side versus demand-side unmet need: Implications for family planning programs. Population and Development Review 2022; https://doi.org/10.1111/padr.12478.

23. Speizer IS, Bremner J, Farid S, et al. Improving family planning indicators of unmet need and demand satisfied. Global Health Science and Practice 2022;10(1): e2100450; https://doi.org/10.9745/GHSP-D-21-00450.

24. Pandey A, Singh KK. Contraceptive use before first pregnancy by women in India (2005–2006): determinants and differentials. BMC Public Health. 2015; 15, 1316. https://doi.org/10.1186/s12889-015-2652-y.

25. Coll CVN, Ewerling F, Hellwig F, de Barros AJD. Contraception in adolescence: The influence of parity and marital status on contraceptive use in 73 low-and middle-income countries. Reproductive Health. 2019; 16, 21. https://doi.org/10.1186/s12978-019-0686-9.

For peer review only - http://bmjopen.bmj.com/site/about/guidelines.xhtml

26. Achyut P, Mishra A, Montana L, Sengupta R, Calhoun LM, Nanda P. Integration of family planning with maternal health services: An opportunity to increase postpartum modern contraceptive use in urban Uttar Pradesh, India. Journal of Family Planning and Reproductive Health Care. 2016;42:107-115.

For peer review only

For peer review only - http://bmjopen.bmj.com/site/about/guidelines.xhtml

Declarations

Author affiliations:
[1]Carolina Population Center, University of North Carolina at Chapel Hill, Chapel Hill, North Carolina, USA
[2]Department of Maternal and Child Health, Gillings School of Global Public Health, University of North Carolina at Chapel Hill, Chapel Hill, North Carolina, USA
[3]Population Council, New Delhi, India
[4]Bill & Melinda Gates Foundation, Seattle, Washington and New Delhi, India

Contributors: ISS conceptualized the study and performed data analyses; AJFZ provided analysis inputs, including coding of some key variables; AJFZ, LMC, PN, NS and GW provided insights into the study design and analysis approach. All authors reviewed drafts of the paper and approved the final version of the paper.

Funding: This research uses data from the study on "Understanding the Lives of Adolescents and Young Adults (UDAYA) in Bihar and Uttar Pradesh" which was collected by the Population Council. Data collection was funded by Bill & Melinda Gates Foundation (OPP1111281) and the David and Lucile Packard Foundation (# 2014-40467). This work was also supported by the Bill & Melinda Gates Foundation (INV-009814). Under the grant conditions of the Foundation, a Creative Commons Attribution 4.0 Generic License has already been assigned to the Author Accepted Manuscript version that might arise from this submission. We also received general support from the Population Research Infrastructure Program through an award to the Carolina Population Center (P2C HD050924) at The University of North Carolina at Chapel Hill.

Competing interests: None declared

Patient consent for publication: Not required

Ethics approval: For the primary data collection, ethics approval was received by the Institutional Review Board at the Population Council (protocol number is 698). All individuals provided consent (written was optional) to participate. This study uses secondary de-identified data and was deemed exempt from ethical review by the University of North Carolina Institutional Review Board (protocol number 21-2643).

Data availability statement: Data are available upon request through the following links:

For UDAYA wave 1 data:
https://dataverse.harvard.edu/dataset.xhtml?persistentId=doi:10.7910/DVN/RRXQNT

For UDAYA wave 2 data
https://dataverse.harvard.edu/dataset.xhtml?persistentId=doi:10.7910/DVN/ZJPKW5

For peer review only - http://bmjopen.bmj.com/site/about/guidelines.xhtml

STROBE Statement—checklist of items that should be included in reports of observational studies

	Item No	Recommendation	Page No
**Title and abstract**	1	(*a*) Indicate the study's design with a commonly used term in the title or the abstract	1
		(*b*) Provide in the abstract an informative and balanced summary of what was done and what was found	1
**Introduction**			
Background/rationale	2	Explain the scientific background and rationale for the investigation being reported	3-4
Objectives	3	State specific objectives, including any prespecified hypotheses	5
**Methods**			
Study design	4	Present key elements of study design early in the paper	5
Setting	5	Describe the setting, locations, and relevant dates, including periods of recruitment, exposure, follow-up, and data collection	5
Participants	6	(*a*) *Cohort study*—Give the eligibility criteria, and the sources and methods of selection of participants. Describe methods of follow-up<br>*Case-control study*—Give the eligibility criteria, and the sources and methods of case ascertainment and control selection. Give the rationale for the choice of cases and controls<br>*Cross-sectional study*—Give the eligibility criteria, and the sources and methods of selection of participants	5
		(*b*) *Cohort study*—For matched studies, give matching criteria and number of exposed and unexposed<br>*Case-control study*—For matched studies, give matching criteria and the number of controls per case	5
Variables	7	Clearly define all outcomes, exposures, predictors, potential confounders, and effect modifiers. Give diagnostic criteria, if applicable	6-10
Data sources/ measurement	8*	For each variable of interest, give sources of data and details of methods of assessment (measurement). Describe comparability of assessment methods if there is more than one group	*6-10*
Bias	9	Describe any efforts to address potential sources of bias	6
Study size	10	Explain how the study size was arrived at	NA (secondary data)
Quantitative variables	11	Explain how quantitative variables were handled in the analyses. If applicable, describe which groupings were chosen and why	6-10
Statistical methods	12	(*a*) Describe all statistical methods, including those used to control for confounding	12
		(*b*) Describe any methods used to examine subgroups and interactions	
		(*c*) Explain how missing data were addressed	8
		(*d*) *Cohort study*—If applicable, explain how loss to follow-up was addressed	

For peer review only

Case-control study—If applicable, explain how matching of cases and controls was addressed<br>*Cross-sectional study*—If applicable, describe analytical methods taking account of sampling strategy	
(*e*) Describe any sensitivity analyses	

Continued on next page

For peer review only

**Results**			
Participants	13*	(a) Report numbers of individuals at each stage of study—eg numbers potentially eligible, examined for eligibility, confirmed eligible, included in the study, completing follow-up, and analysed	13, Table 1
		(b) Give reasons for non-participation at each stage	
		(c) Consider use of a flow diagram	
Descriptive data	14*	(a) Give characteristics of study participants (eg demographic, clinical, social) and information on exposures and potential confounders	Table 1
		(b) Indicate number of participants with missing data for each variable of interest	
		(c) *Cohort study*—Summarise follow-up time (eg, average and total amount)	
Outcome data	15*	*Cohort study*—Report numbers of outcome events or summary measures over time	*13-18; Table 1*
		Case-control study—Report numbers in each exposure category, or summary measures of exposure	
		Cross-sectional study—Report numbers of outcome events or summary measures	
Main results	16	(*a*) Give unadjusted estimates and, if applicable, confounder-adjusted estimates and their precision (eg, 95% confidence interval). Make clear which confounders were adjusted for and why they were included	13-18; Tables 2-4
		(*b*) Report category boundaries when continuous variables were categorized	
		(*c*) If relevant, consider translating estimates of relative risk into absolute risk for a meaningful time period	
Other analyses	17	Report other analyses done—eg analyses of subgroups and interactions, and sensitivity analyses	13-18; Table 5
**Discussion**			
Key results	18	Summarise key results with reference to study objectives	18-19
Limitations	19	Discuss limitations of the study, taking into account sources of potential bias or imprecision. Discuss both direction and magnitude of any potential bias	20-21
Interpretation	20	Give a cautious overall interpretation of results considering objectives, limitations, multiplicity of analyses, results from similar studies, and other relevant evidence	18-19
Generalisability	21	Discuss the generalisability (external validity) of the study results	18-19
**Other information**			
Funding	22	Give the source of funding and the role of the funders for the present study and, if applicable, for the original study on which the present article is based	27

*Give information separately for cases and controls in case-control studies and, if applicable, for exposed and unexposed groups in cohort and cross-sectional studies.

Note: An Explanation and Elaboration article discusses each checklist item and gives methodological background and published examples of transparent reporting. The STROBE checklist is best used in conjunction with this article (freely available on the Web sites of PLoS Medicine at http://www.plosmedicine.org/, Annals of Internal Medicine at http://www.annals.org/, and Epidemiology at http://www.epidem.com/). Information on the STROBE Initiative is available at www.strobe-statement.org.

For peer review only

For peer review only - http://bmjopen.bmj.com/site/about/guidelines.xhtml

BMJ Open

Longitudinal examination of young married women's fertility and family planning intentions and how they relate to subsequent family planning use in Bihar and Uttar Pradesh India

Journal:	*BMJ Open*
Manuscript ID	bmjopen-2022-064487.R1
Article Type:	Original research
Date Submitted by the Author:	24-Jun-2022
Complete List of Authors:	Speizer, Ilene; University of North Carolina at Chapel Hill Gillings School of Global Public Health, Maternal and Child Health<br>Zavier, A.J.; Population Council India<br>Calhoun, Lisa; University of North Carolina at Chapel Hill, Carolina Population Center,<br>Nanda, Priya; Bill and Melinda Gates Foundation India<br>Saggurti, Niranjan; Population Council<br>Hainsworth, Gwyn; Bill & Melinda Gates Foundation
**Primary Subject Heading**:	Sexual health
Secondary Subject Heading:	Global health, Public health
Keywords:	PUBLIC HEALTH, STATISTICS & RESEARCH METHODS, REPRODUCTIVE MEDICINE

SCHOLARONE™
Manuscripts

I, the Submitting Author has the right to grant and does grant on behalf of all authors of the Work (as defined in the below author licence), an exclusive licence and/or a non-exclusive licence for contributions from authors who are: i) UK Crown employees; ii) where BMJ has agreed a CC-BY licence shall apply, and/or iii) in accordance with the terms applicable for US Federal Government officers or employees acting as part of their official duties; on a worldwide, perpetual, irrevocable, royalty-free basis to BMJ Publishing Group Ltd ("BMJ") its licensees and where the relevant Journal is co-owned by BMJ to the co-owners of the Journal, to publish the Work in this journal and any other BMJ products and to exploit all rights, as set out in our licence.

The Submitting Author accepts and understands that any supply made under these terms is made by BMJ to the Submitting Author unless you are acting as an employee on behalf of your employer or a postgraduate student of an affiliated institution which is paying any applicable article publishing charge ("APC") for Open Access articles. Where the Submitting Author wishes to make the Work available on an Open Access basis (and intends to pay the relevant APC), the terms of reuse of such Open Access shall be governed by a Creative Commons licence – details of these licences and which Creative Commons licence will apply to this Work are set out in our licence referred to above.

Other than as permitted in any relevant BMJ Author's Self Archiving Policies, I confirm this Work has not been accepted for publication elsewhere, is not being considered for publication elsewhere and does not duplicate material already published. I confirm all authors consent to publication of this Work and authorise the granting of this licence.

For peer review only

Longitudinal examination of young married women's fertility and family planning intentions and how they relate to subsequent family planning use in Bihar and Uttar Pradesh India

Ilene S. Speizer,[1,2*] A. J. Francis Zavier,[3] Lisa M. Calhoun,[1] Priya Nanda,[4] Niranjan Saggurti,[3] Gwyn Hainsworth[4]

*Corresponding author: Ilene S. Speizer, Department of Maternal and Child Health, Gillings School of Global Public Health, University of North Carolina at Chapel Hill, Chapel Hill, NC, USA. ilene_speizer@unc.edu

Abstract

Objectives: This study examines which fertility and family planning (FP) intentions are related to subsequent FP use in a sample of young, married women in India.

Design: We use three-year longitudinal data from married women ages 15-19 in 2015-16 (Wave 1) who are not using contraception to examine factors associated with any use of FP in 2018-19 (Wave 2).

Setting: Data were collected in the states of Bihar and Uttar Pradesh, India.

Participants: A representative sample of 4,893 young married women ages 15-19 was surveyed in 2015-16 and 4,000 of them were found and interviewed three years later. This analysis focused on the 3,614 young women who were not using FP at Wave 1.

Primary outcomes: This study examines FP use at Wave 2 as the main outcome variable.

Results: Multivariate analyses demonstrated that young women who wanted to delay childbearing three or more years or who did not want any(more) children at Wave 1 were more likely to use contraception at Wave 2. Additionally, intention to use FP in the next 12 months at Wave 1 was significantly associated with FP use at Wave 2 whereas unmet need at Wave 1 was not significantly related to subsequent use. A combined measure of fertility desires and intention to use FP demonstrated the importance of both measures on subsequent use. Having any children and being pregnant at Wave 1 were both related to FP use at Wave 2.

Conclusions: It is important to reach young, married women prior to a first pregnancy with nuanced messages addressing their fertility and FP intentions. Programs targeting women at antenatal and

For peer review only - http://bmjopen.bmj.com/site/about/guidelines.xhtml

postpartum visits are important for young women to help support them to use FP to address their desires to delay or limit future childbearing for the health and well-being of themselves and their children.

For peer review only

For peer review only - http://bmjopen.bmj.com/site/about/guidelines.xhtml

Strengths and limitations of this study

- Longitudinal data permitted identifying meaningful measures of fertility desires and family planning use intentions for young, married women.
- The results from this study can be used to better inform program targeting of the women with the greatest contraceptive needs.
- Findings demonstrated that recently married young women need information and services (at the time of marriage, prior to a birth, or at antenatal, or postpartum care) to affect their agency and intentions to use contraception in the future.
- The study was limited because a key measure, intention to use family planning in the next 12 months, did not correspond to the three-year follow-up period.
- Some women may have adopted a method between the two survey waves, and this was not captured with these data; this was a limitation of this analysis.

For peer review only

For peer review only - http://bmjopen.bmj.com/site/about/guidelines.xhtml

Longitudinal examination of young married women's fertility and family planning intentions and how they relate to subsequent family planning use in Bihar and Uttar Pradesh India

Introduction

Early and closely spaced pregnancies can have long term negative health and well-being outcomes for young mothers and their children [1]. In places where the age at marriage is low and gender norms dictate immediate childbearing post marriage, understanding factors associated with young women's (and couples') use of family planning (FP) to delay and space births is helpful for understanding how to develop programs that lead to improved individual, maternal, and child health outcomes.

At the aggregate level, unmet need for FP is a policy variable, demonstrating hypothetical gaps between fertility desires and contraceptive use. Unmet need is defined as the population of fecund, sexually active women of reproductive age who report a desire to avoid childbearing 2+ years or not have any(more) children and are not using contraception [2]. Recent studies that examine the association between unmet need, fertility desires, and subsequent fertility and FP behaviors demonstrate that there is a weak link between unmet need and outcomes [3-5]. In a study using longitudinal data from Uganda, Sarnak and colleagues [3] demonstrate that among non-contracepting women in 2014, those with an unmet need are slower to adopt a contraceptive method over the four-year follow-up period than those without an unmet need. The authors also find that intentions to use contraception in the future are significantly related to contraceptive adoption [3]. Importantly, using a combined measure of unmet need and intentions to use contraception in the future, women who intend to use, whether or not they have an unmet need, have a shorter time to adoption in the follow-up period. Further, in a study among urban women from six cities in Uttar Pradesh, India, Speizer and colleagues [5] demonstrate that in a two-year follow-up period, women who did not want any(more) children were significantly less likely to have a birth than all other women; this effect was bigger among

For peer review only - http://bmjopen.bmj.com/site/about/guidelines.xhtml

those using contraception at baseline. However, no difference in birth experience is found between women who wanted to space and women who wanted a pregnancy soon, no matter their baseline contraceptive use. These results are consistent with an earlier study from India by Roy and colleagues [6] that found that over a six-year follow-up period, many women did not adhere to their fertility preferences nor to their FP use intentions.

Recently, colleagues have proposed the importance of considering intention to use contraception in addition to unmet need to better assess actual gaps in FP use [7,8]. Moreau and colleagues [7] propose a measure called current status unmet demand that removes currently pregnant and recently postpartum women and incorporates women's intentions to use contraception in the future. In aggregate estimations of women of all ages across 46 countries, adjusted unmet need is lower (13.8%) than the standard unmet need (16.4%) and only 6.7% of women have an unmet need and intend to use and 7.2% have an unmet need but do not intend to use in the future [7]. Similarly, using data from India, Panda and colleagues [8] identify those women of all ages who have an unmet need and intend to use FP in the next 12 months and demonstrate that this group is important for identifying women most in need of FP.

Prior studies focus on all women of reproductive ages (ages 15-49). Less is known about the fertility desires and FP use intentions of adolescents (ages 15-19); what is known among young women typically comes from cross-sectional studies [9-13]. Young women are of particular interest since they are at the beginning of their reproductive lives and some may want to delay a first birth or space births to pursue their aspirations (e.g., stay in school, work, get to know their husbands) or for better maternal and child health outcomes. It is important to examine young women in the India context where at the time of the 2019-2021 National Family Health Survey (NFHS) 16% of women ages 20-24 in Uttar Pradesh [14] and 41% of their counterparts from Bihar [15] were married before the legal minimum age of marriage of 18; these young married women have often already begun childbearing and thus may have underserved FP needs.

For peer review only

This study uses longitudinal data collected among adolescents from Bihar and Uttar Pradesh, India to examine how fertility desires and FP use intentions among married adolescents evolve over the adolescent and young adult years. We also determine the utility of various measures of fertility desires, unmet need, and FP intentions for supporting programs to identify young women who have the greatest need for FP and are the most likely to adopt FP in the future.

Methods

Study sample

Data for this study came from two states in northern India – Bihar and Uttar Pradesh. Both states have large populations, are among the poorest in the country, and are predominately rural [16]. This study used secondary data collected as part of a study led by the Population Council in India called "Understanding the Lives of Adolescent and Young Adults," also called the UDAYA study. In 2015-2016, Wave 1 data were collected among a representative sample of unmarried girls and boys (ages 10-19) and married girls ages 15-19 [17]. More details on the study design can be found elsewhere [18,19] and on the project website (www.projectudaya.in). Three years later (between 2018-2019), Wave 2 follow-up data were collected with the sample of participants who were interviewed in Wave 1 [20].

This analysis focused on the sample of women ages 15-19 who were married with Gauna performed (i.e., the marriage is consummated) at Wave 1 who were followed in Wave 2 and had information on the fertility and FP variables of focus in this analysis. The full sample of young women surveyed in Wave 1 was 4,893 (see Table 1). A total of 4,000 of these women were re-interviewed in Wave 2 (81.7%) while 893 women (18.3%) were not found at Wave 2. Table 1 presents the characteristics of the full sample, the analysis sample (n=4,000) and the sample lost-to-follow-up. The women who were not interviewed at Wave 2 tended to be from Uttar Pradesh (p<0.001), have no births at Wave 1 (p=0.10), and be from urban areas (p<0.001). In the analysis of which young married women were using FP at Wave 2, the

For peer review only - http://bmjopen.bmj.com/site/about/guidelines.xhtml

sample was limited to married adolescents who were non-users at Wave 1 (n=3,616) and had full information on the explanatory variables (n=3,614).

Outcome variable

The key outcome variable was use of any contraceptive method at Wave 2. Women who at Wave 2 reported using any modern method (pill, IUD, injectable, implant, condom, diaphragm, foam/jelly, female or male sterilization, female condom, or LAM)[a] or any traditional method (rhythm method and withdrawal) were coded 1. All others were coded zero, including those who were pregnant at Wave 2 and those who reported never using a method at Wave 2. Among the full sample at Wave 2 (n=4,000), 17% were using any method and 10% were using a modern method. The analyses presented here focused on any method use; however, models were also run with the outcome of modern method use and the results were similar.

[a] The list of modern methods is the same as used in large surveys such as the Demographic and Health Survey and by the FP2030 Initiative to measure the core indicators. The only difference was that emergency contraception was not asked among the methods that women could report using in the UDAYA survey.

For peer review only - http://bmjopen.bmj.com/site/about/guidelines.xhtml

Table 1. Descriptive information on sample of married[a] participants at Wave 1 and the longitudinal sample for analysis

	Full sample at Wave 1[a] (n=4,893)	Wave 2 follow-up sample (n=4,000; 81.7%)	No follow-up sample (n=893; 18.3%)	p-value*
**State (%)**				
Uttar Pradesh	60.4	58.1	71.0	<0.001
Bihar	39.6	42.0	29.0	
**Age in years (%)**				
15	1.9	1.8	2.5	NS
16	6.7	6.9	5.8	
17	15.6	15.6	15.6	
18	32.5	32.6	31.8	
19	43.3	43.1	44.3	
**Parity (%)**				
None	61.0	59.9	66.0	0.10
1	31.8	32.3	29.2	
2+	7.2	7.8	4.8	
**Education (%)**				
None	24.8	24.2	27.6	NS
1-4 years	5.9	5.7	7.0	
5-7 years	16.7	16.7	17.0	
8-9 years	26.4	27.0	23.8	
10-11 years	12.7	12.7	12.6	
12+ years	13.4	13.7	12.2	
**Wealth (%)**				
Lowest	15.2	14.8	16.9	NS
Low	20.8	21.3	18.8	
Middle	23.5	23.3	24.6	
High	23.7	24.0	22.5	
Highest	16.7	16.6	17.3	
**Religion (%)**				
Hindu	82.3	83.0	79.0	NS
Muslim	17.5	16.8	20.7	
Others	0.2	0.2	0.3	
**Caste (%)**				
Scheduled Castes/Tribes	30.0	29.7	31.1	NS
Other Backward Castes	57.5	58.1	54.8	
General	12.5	12.2	14.1	
**Residence (%)**				
Urban	10.8	9.7	16.1	<0.001
Rural	89.2	90.3	83.9	

All values weighted using Wave 1 weights. *F-test comparing analysis sample to those lost to follow-up.
[a]Sample that was married with Gauna performed.

For peer review only - http://bmjopen.bmj.com/site/about/guidelines.xhtml

Key independent variables

To understand fertility desires and FP use intentions of young married women, we examined whether variables from Wave 1 were associated with use of FP at Wave 2, among non-users at Wave 1. First, we examined Wave 1 desire for a future pregnancy and the timing of that desire (see Table 2 for these variables). This was categorized as: wants a pregnancy before 2 years (soon), wants in 2 years, wants in 3 years, wants in 4+ years, undecided, and wants no more children. Second, we included the standard measure of unmet need (no vs. yes), calculated based on questions about fertility desires, FP use, and intentionality of a recent/last pregnancy as traditionally defined [2]. We also included two questions on intention to use FP – one about intention to use in the next 12 months, and the other about intention to ever use in the future; both of these were coded as no, yes, or don't know. For the intention to use FP questions, women who had ever used a method and were current non-users were not asked this question (n=75); for this analysis, these women were coded as intends to use based on their prior experience. In models that dropped the women with missing information, the results were like those presented. In this Indian context where the norm is to have 2-3 children and then get sterilized, it was not surprising that most young women intended to ever use in the future (81% at Wave 1); therefore, for this analysis, we focused on intention to use in the next 12 months, even though the follow-up period is 3 years.

For peer review only - http://bmjopen.bmj.com/site/about/guidelines.xhtml

Table 2. Fertility and family planning variables at Wave 1 and Wave 2 of follow-up sample of women ages 15-19 and married at Wave 1

	Wave 1<br>%	Wave 2<br>%
**Desire for a pregnancy**		
Wants within 2 years	31.3	20.8
Wants in 2 years	18.4	12.5
Wants in 3 years	15.0	8.9
Wants in 4+ years	12.8	8.0
Undecided	10.2	8.3
Wants no more	12.3	41.4
**Currently using any method**	n=3,994*	
No	91.0	82.8
Yes	9.0	17.2
**Unmet need**		n=3,965**
No	51.4	54.3
Yes	48.6	45.7
**Currently pregnant**		
No	78.8	83.2
Yes	21.2	16.8
**Intention to use FP ever in the future**	n=3,998*	
Yes	81.3	89.8
No	14.4	9.1
Don't know	4.3	1.2
**Intention to use FP in next 12 months**	n=3,998*	
Yes	48.2	62.2
No	46.1	36.9
Don't know	5.7	0.9
**Experience birth or pregnancy between Waves (includes currently pregnant at Wave 2)**	NA	
No		13.32
Yes		86.7
**Intentionality of most recent birth or pregnancy experienced**	NA	n=3,461
Wanted then		65.6
Wanted later		31.2
Wanted no more		3.2

Unweighted number of observations shown was 4,000 unless shown otherwise. * Two women missing information on this measure at Wave 1. **35 women missing relevant information for Wave 2 unmet need measure. Wave 1 and Wave 2 values used Wave 2 weights that adjusted for loss to follow-up.

For peer review only - http://bmjopen.bmj.com/site/about/guidelines.xhtml

In response to recommendations by Moreau and colleagues [7] and Panda and colleagues [8] we created measures that jointly captured fertility desires and intentions to use contraception in the future. First, we created a combined measure based on fertility desires and intentions to use FP in the future with six distinct categories (see Table 3). Second, we created a combined measure based on unmet need and intention to use FP in the future; this variable had four categories that can be seen in Table 3. All women who reported that they did not know if they intended to use in the next 12 months were grouped with "no intention to use."

For peer review only

For peer review only - http://bmjopen.bmj.com/site/about/guidelines.xhtml

Table 3. Fertility and family planning variables of non-users with full information at Wave 1 and the correlation with Wave 2 family planning use

Wave 1 characteristic	Wave 1 non-users %	Percent using FP at Wave 2	
**Desire for a pregnancy**			
Wants within 2 years	33.0	7.9	p<0.001
Wants in 2 years	18.6	12.5	
Wants in 3 years	14.3	16.5	
Wants in 4+ years	11.8	16.6	
Undecided	11.0	13.9	
Wants no more	11.4	26.2	
**Unmet need**			
No	46.6	11.6	p=0.006
Yes	53.4	15.6	
**Intention to use FP in next 12 months**			
Yes	43.0	17.6	p<0.001
No	50.8	9.9	
Don't know	6.3	18.5	
**Fertility desire†/FP use intention‡**			
Wants now/no intention	32.2	9.1	p=0.001
Wants now/intention to use	11.7	10.1	
Wants later/no intention	20.2	11.0	
Wants later/intention to use	24.5	18.0	
Wants no more/no intention	4.6	22.3	
Wants no more/intention to use	6.8	28.8	
**Unmet need/FP use intention‡**			
No unmet need/no intention	30.7	9.8	p=0.001
No unmet need/intention to use	15.9	15.3	
Unmet need/no intention to use	26.3	12.1	
Unmet need/intention to use	27.1	19.0	

This table focuses on the non-users of any method at Wave 1 (n=3,614). All percentages weighted using Wave 2 weights. Significance testing determined if family planning use differed by the various Wave 1 fertility desire and intention to use categories. † Wants now included those wanting in two years or undecided; wants later was those who want to delay 2+ years. ‡No intention included those who were undecided on future use.

We also descriptively examined if women experienced a birth between Wave 1 and Wave 2 (or were currently pregnant at Wave 2) and the intentionality of the recent birth or current pregnancy as

For peer review only - http://bmjopen.bmj.com/site/about/guidelines.xhtml

reported at Wave 2. This information was useful for better understanding the utility of assessing fertility desires among young, married women in Bihar and Uttar Pradesh and the fluidity of these desires.

Demographic variables

Because fertility desires and contraceptive behaviors are closely tied to a woman's current pregnancy status, we adjusted for whether the woman was currently pregnant at Wave 1 (no vs. yes); this avoided dropping currently pregnant women from the analyses. We also adjusted for Wave 1 state (Bihar vs. Uttar Pradesh), caste (Scheduled Caste, Scheduled Tribe, Other Backward Caste, General), age in years, parity (no children, one child, two or more), education level (none, primary, 8-9 years of education, 10+ years of education), religion (Hindu vs. all others), wealth group (lowest, low, medium, high, highest), and place of residence (urban vs. rural). The coding and distribution of these variables is shown in Table 1.

Analysis

Univariate and bivariate analyses were used to examine the associations between fertility desires and FP use intentions at Wave 1 and Wave 2 contraceptive use. All descriptive statistics that examined the characteristics of the Wave 1 sample, including examination of loss to follow-up, used Wave 1 weights. All analyses that focused on the longitudinal sample used Wave 2 weights to adjust for loss to follow-up. Multivariate logistic regression analyses were used to inform which young non-users at Wave 1 were the most likely to use any FP method at Wave 2, adjusting for Wave 1 demographic characteristics. Recognizing that some of the young women were pregnant at Wave 1 and did not need FP at that time; instead of dropping them as typically done, we controlled for this Wave 1 pregnancy to see how this related to FP use at Wave 2. All multivariate analyses were unweighted but adjusted for sample clustering. This study used secondary de-identified data and was deemed exempt from ethical review by the University of North Carolina Institutional Review Board.

For peer review only - http://bmjopen.bmj.com/site/about/guidelines.xhtml

Patient and public involvement: Patients and/or the public were not involved in the design or conduct or reporting or dissemination plans of this research.

Results

Table 1 shows that the sample of married adolescents was predominately ages 18 and 19 at Wave 1 (mean age is 18.0 years); this is consistent with the legal age of marriage for women in India. By Wave 2, the cohort was ages 17-23 with a mean age of 20.8 years. More than half of the sample had no children and one third had one child; the remaining women (about 8%) had two or more children by the time of the Wave 1 survey. About a quarter of the sample had no education while about half had 8+ years of education. The majority of the sample was Hindu, and most participants lived in rural areas. Finally, more than half of the sample was Other Backward Castes and a quarter were Scheduled Castes/Tribes.

Table 2 presents a comparison of the fertility and FP variables for the longitudinal sample; all results used the Wave 2 weights. At Wave 1, a third of women wanted a child soon (within 2 years) and another third wanted a child in 2 or 3 years. Nearly 10% of women were undecided about future childbearing. Thirteen percent of women reported that they wanted to wait 4+ years before having a child (or another child) and 12% said they do not want (any)more children. By Wave 2, a greater percentage of women wanted no more children as many had given birth between waves (87% experienced a pregnancy or birth). Only 9% of this young sample was using any method of contraception at Wave 1 and 48.6% had an unmet need for FP. At Wave 1, one-fifth of the sample was currently pregnant; these women may have a future need for FP, following the delivery of their baby.

Also included in Table 2 is the intention to use FP (ever or in the next 12 months). In this India context where sterilization is common, it is not surprising that more than four-fifths intended to use FP ever in the future. Yet only 48% of these young women intended to use FP in the next 12 months.

For peer review only - http://bmjopen.bmj.com/site/about/guidelines.xhtml

Table 2 includes the same measures at Wave 2 to examine aggregate changes in the three-year follow-up period. As mentioned above, 87% of women had a birth between waves or were currently pregnant at the time of the Wave 2 survey. Two-thirds of the births/pregnancies were wanted then and one third were wanted "later," that is the woman wanted to wait 2+ years before the birth. This high birth experience between waves affected the other fertility and FP measures assessed at Wave 2. Notably, while contraceptive use increased between waves (9% to 17%), it did not increase as much as the desire to avoid pregnancy (12% to 41%).

Table 3 focuses on the non-users of contraception at Wave 1 and presents their fertility and FP variables as well as how this corresponded to FP use at Wave 2. Based on Wave 1 fertility desires, those young women who wanted no more children were more likely to be using by Wave 2 (27%) than all other fertility desire groups; this was followed by those who wanted to wait 3+ years before the next birth. Based on unmet need at Wave 1, a significantly greater percentage of those with an unmet need at Wave 1 were using at Wave 2 (16%) compared to those without an unmet need at Wave 1 (12%); however, this difference was smaller than would be expected given the importance assigned to this global indicator. As expected, a significantly greater percentage of young women who reported at Wave 1 that they intended to use FP in the next 12 months were using at Wave 2 compared to those who said they did not intend to use in the next 12 months; those who were unsure if they will use were also more likely to use.

Table 3 also includes a combined fertility desire and intention to use variable. This combined variable showed that both fertility desires and FP use intentions matter in the expected directions such that those who wanted to avoid or delay childbearing were more likely to be using at Wave 2, especially if they intended to use in the next year. For the combined unmet need and intention to use measure we found significant differences between the groups; however, there was less variability in use between the unmet need/intention to use groups than when fertility desires were combined with FP use intentions.

For peer review only - http://bmjopen.bmj.com/site/about/guidelines.xhtml

Table 4 presents the multivariable logistic regression odds ratios (OR) and 95% confidence intervals (CI) for the analysis of Wave 2 use of any contraception among non-users at Wave 1, including Wave 1 fertility desires and FP intentions and adjusting for Wave 1 demographics. Model 1 shows that women who at Wave 1 wanted to wait 4+ years (OR: 1.68; 95% CI: 1.19-2.37) and those women who did not want any(more) children (OR: 2.27; 95% CI: 1.58-3.25) were significantly more likely to be using a method at Wave 2 than women who wanted a child within 2 years. In addition, women who wanted to wait 3 years were significantly more likely to be using at Wave 2. No difference was found in Wave 2 use between women who were undecided about future childbearing and women who wanted a child in two years as compared to women who at Wave 1 wanted a child soon. Also included in this model was if the woman was pregnant at Wave 1; those women who were pregnant were significantly more likely to use at Wave 2 than those who were not pregnant. Further, women who had no children at Wave 1 were significantly less likely to use at Wave 2 and those with 2+ children were significantly more likely to use at Wave 2 compared to women with one child. The other Wave 1 demographics (age, education, caste, religion, place of residence, and wealth quintile) were in the expected directions. Findings for Wave 1 pregnancy, parity and the demographics are consistent across all models shown (contact first author for these results).

For peer review only - http://bmjopen.bmj.com/site/about/guidelines.xhtml

Table 4. Examination of use of any contraception at Wave 2 based on Wave 1 characteristics and Wave 1 fertility desires/FP intentions (among non-users at Wave 1, n=3,614)

	Model 1	Model 2	Model 3	Model 4	Model 5
	OR (95% CI)	OR (95% CI)	OR (95% CI)	OR (95% CI)	OR (95% CI)
Fertility Desires					
Wants within 2 years (ref)	1.0				
Wants in 2 years	1.17 (0.83-1.66)				
Wants in 3 years	1.54 (1.08-2.20)*				
Wants in 4+ years	1.68 (1.19-2.37)**				
Undecided	1.33 (0.90-1.97)				
Wants no more	2.27 (1.58-3.25)***				
Intention to use FP in 12 months					
No (ref)		1.0			
Yes		1.33 (1.08-1.64)**			
Don't know		1.32 (0.87-2.01)			
Unmet need					
No (ref)			1.0		
Yes			1.16 (0.96-1.40)		
Fertility desire/FP use intention					
Wants now/no intention (ref)				1.0	
Wants now/intention to use				0.98 (0.68-1.42)	
Wants later/no intention				1.08 (0.82-1.42)	
Wants later/intention to use				1.42 (1.08-2.64)*	
Wants no more/no intention				1.69 (1.08-2.64)*	
Wants no more/intention to use				2.20 (1.55-3.13)***	
Unmet need/FP use intention					
No unmet/no intention (ref)					1.0
No unmet/intention					1.36 (1.01-1.83)*
Unmet need/no intention					1.19 (0.92-1.55)
Unmet need/intention					1.43 (1.09-1.86)**

Note: All models also controled for Wave 1 pregnancy status, Wave 1 parity, state, caste, age, education, religion, place of residence, and wealth; +p≤0.10; *p≤0.05; ***p≤0.001. Multivariate models were unweighted but adjusted for sample clustering.

For peer review only - http://bmjopen.bmj.com/site/about/guidelines.xhtml

Model 2 presents the same model with intention to use contraception in the next 12 months as the key variable. Adjusting for demographic factors, those women who at Wave 1 intended to use FP soon were significantly more likely to use at Wave 2 (OR: 1.33; 95% CI: 1.08-1.64) than those who did not intend to use FP in the next year.

Model 3 includes the unmet need measure at Wave 1. There was no difference in use at Wave 2 between those with no unmet need and those with an unmet need at Wave 1.

Model 4 includes the combined fertility desire and intention to use FP variable. Those women who wanted to avoid childbearing at Wave 1 were the most likely to use, no matter their Wave 1 intention to use FP. In addition, those women who wanted to delay childbearing 2+ years and who reported an intention to use in the next 12 months were significantly more likely to be using at Wave 2 than their counterparts who wanted a child soon and did not intend to use. No difference in use at Wave 2 was found between those who wanted to delay childbearing 2+ years but did not intend to use and those who wanted a child soon and do not intend to use.

Finally, Model 5 includes the combined unmet need and intention to use FP measure. Those women who had an unmet need and intended to use FP in the next 12 months had higher odds of using (OR: 1.43; 95% CI: 1.09-1.86) than those women with no unmet need and no intention to use. Further, women who at Wave 1 had no unmet need and an intention to use were also more likely to use at Wave 2 (OR: 1.36; 95% CI: 1.01-1.83) than those with no unmet need and no intention to use.

Finally, Table 5 provides additional context on how fertility desires among the young women play out in actual behaviors. Among women who at Wave 1 wanted a child in two years or were undecided about future childbearing, over 90% became pregnant or had a birth between waves. Among those who wanted to wait 4+ years or did not want any(more) children, a significantly lower percentage (82% and 81%, respectively) became pregnant or had a birth between waves. Notably, among those who wanted a child soon (within 2 years), only 84% became pregnant; this might be related to sub-fecundity among

For peer review only - http://bmjopen.bmj.com/site/about/guidelines.xhtml

these women who may have been trying to get pregnant for a while. Also shown in Table 5 is the intentionality of experienced pregnancies. While the results were significantly different across groups (p<0.001), it is notable that most births/pregnancies experienced were considered wanted then (58%-73%) or wanted later (26%-38%); only a small percentage of pregnancies were considered unwanted and this was predominately among those who did not want to become pregnant at Wave 1 (10%). This table indicates post-hoc rationalization of pregnancies among these young women who were early in their childbearing years.

Table 5. Pregnancy experience between Waves and intentionality of pregnancy among those who experienced a pregnancy/birth at Wave 2

		Intentionality of birth/pregnancy at Wave 2 (%) (n=3,461)			
**Wave 1: Desire for a pregnancy**	**% had birth or pregnancy between waves**	**Wanted then**	**Wanted later**	**Did not want**	**Total % (n)**
Wants within 2 years	84.5	72.9	26.3	0.9	100 (n=996)
Wants in 2 years	92.2	65.3	32.1	2.7	100 (n=659)
Wants in 3 years	88.7	61.2	37.1	1.7	100 (n=542)
Wants in 4+ years	82.0	57.6	38.4	4.1	100 (n=424)
Undecided	93.4	66.5	28.6	4.9	100 (n=432)
Wants no more	81.0	60.1	30.0	9.9	100 (n=408)
	p<0.001		p<0.001		

Uses Wave 2 weights. For percent that had a birth, n=4,000 women married at Wave 1 that also have Wave 2 data.

Discussion

The descriptive findings from this study demonstrate that over the follow-up period, 87% of the sample had a birth between waves or was pregnant at Wave 2; this suggests the importance of early childbearing among young, married couples from Bihar and Uttar Pradesh. Further, pregnancy/birth experience between waves was associated with Wave 1 fertility desires; however, the majority of pregnancies experienced, no matter the Wave 1 desires, were considered wanted (then or later). Prior

For peer review only - http://bmjopen.bmj.com/site/about/guidelines.xhtml

studies from India have demonstrated that fertility desires of women of all ages (not just adolescents as included here) are fluid and there is post-hoc rationalization of pregnancies/births experienced [5,6,21]. With the data available, it was not possible to know if the women changed their fertility desires between waves, if their partner or someone else influenced their pregnancy experience, if they lacked access to contraception to meet their needs, if they did not have the agency to act on their fertility desires, or if there were other reasons for the inconsistent pregnancy/birth experience in the follow-up period.

From the data available, it appears that there were FP needs in this sample of young women. At Wave 1, only 9% of women were using a contraceptive method; however, unmet need for FP was high (49%). Unmet need in the UDAYA sample was higher than in the 2015/2016 India NFHS where 22.5% of women ages 15-19 in union from Uttar Pradesh [22] and 29.6% of their counterparts from Bihar [23] had an unmet need for FP. Further, while about 40% of these young women reported an intention to use contraception in the next 12 months, FP use only increased by about 8 percentage points between Wave 1 and Wave 2. Whether the gap between intention and actual use reflected fluid adoption and discontinuation of methods in the follow-up period, a lack of demand for methods (e.g., concerns about side effects), or barriers to access to methods when women were in need, was difficult to assess with the data available. A recent paper by Senderowicz and Maloney [24] proposes a strategy to divide unmet need into two categories: supply side versus demand side unmet need; this would help to better assess barriers to use. Further, Panda and colleagues [8] propose that incorporating intention to use FP into unmet need measures, as done here, may help with identifying those women with the most need in India.

In our multivariate analyses, unmet need at Wave 1 was not related to Wave 2 contraceptive use. This is not surprising given that unmet need is meant to be used at a population level and is an investigator determined and not woman-determined assessment of need [2,24,25]. That said, fertility

For peer review only - http://bmjopen.bmj.com/site/about/guidelines.xhtml

desires matter such that those women who reported a desire to delay 3+ years and those women who wanted to avoid childbearing were more likely to use at Wave 2 than those women who wanted a child soon. Intention to use FP in the next 12 months was also related to Wave 2 contraceptive use. The combined fertility desire/intentions to use variable and the unmet need/intentions to use variable were both related to subsequent use. These results inform future measurement of fertility desires and FP intentions among young women. First, unmet need on its own is not a meaningful indicator; it is difficult to calculate, not meant to be used at an individual level, and not useful among young women who are at the beginning of their reproductive careers. That said, understanding young women's fertility desires with a more detailed measure (i.e., more categories than just want soon, want later, and does not want as typically used) was informative for understanding their "plans" for future childbearing, even if they were not able to follow through on these fertility desires. Further, examining young women's intentions to use a method in the next 12 months was also a meaningful and simple indicator that can be used individually or in combination with fertility desires to help inform who is most likely to adopt a method in the future. The combined fertility desires and family planning use intentions measure is simple to measure, often included in counseling discussions, and can be useful for program targeting.

Our analyses demonstrated that women who were currently pregnant at Wave 1 and/or had already had one or two pregnancies by the time of the Wave 1 survey were significantly more likely to use at Wave 2 than all others. This reflects the norm in India that young, married couples should have a birth (or two) prior to using FP [26,27]. FP programs seeking to reach young, married women in India should promote FP messages in the antenatal and postpartum periods to ensure that there are no missed opportunities for provision of FP services [28]. While currently pregnant women do not have a "current need," they do have an imminent need and may be candidates for immediate postpartum contraception, including long-acting reversible contraception.

For peer review only - http://bmjopen.bmj.com/site/about/guidelines.xhtml

This study is not without limitations. An important limitation is that the intention to use FP in the next 12 months variable does not correspond to the three-year follow-up period. Given the high intention to use ever in the future, the 12-month variable seemed more relevant for this analysis. Second, we were not able to examine the timing of adoption in the follow-up period given that calendar data were not available. Further, it was not possible to examine if women adopted and discontinued in the follow-up period to meet their fertility desires. Future studies that include calendar data on timing of adoption/use of FP as part of longitudinal data are important to overcome these limitations.

Conclusion

Young women's fertility desires and family planning use intentions can be used by family planning programs to identify those young women with the most need for family planning and to develop programs that support fertility transitions over the early reproductive years. In this study, the women who were the least likely to adopt a FP method by Wave 2 reported at Wave 1 that they wanted a child soon, did not have any children, and/or did not intend to use in the future. Programs targeting these young women should focus on demand side programming to help build young women's agency to act on their family, fertility and life course desires. This could happen through outreach or mass media programs that have messages about the benefits of FP for couple relationship strengthening, school and work attainment, as well as for the health of the mother and the child. These types of demand creation programs may address gaps in knowledge and access, increase intention to use in the future, and increase the agency of women to seek a method when or if they so desire. This may require training frontline workers on having conversations about FP with young, recently married women, their male partners and/or other gatekeepers such as mothers-in-law as well as training them to provide contraceptive methods or refer those who want to use a method they do not offer. Over time,

For peer review only - http://bmjopen.bmj.com/site/about/guidelines.xhtml

increased engagement with young women may lead to delayed first births and better spacing between young women's births and lead to better maternal and child health and well-being.

For peer review only

For peer review only - http://bmjopen.bmj.com/site/about/guidelines.xhtml

References

1. Norton M, Chandra-Mouli V, Lane C. Interventions for preventing unintended, rapid repeat pregnancy among adolescents : A review of the evidence and lessons from high-quality evaluations. Global Health Science and Practice. 2017; 5(4): 547-570.
2. Bradley SEK and Casterline JB. Understanding unmet need: History, theory, and measurement. Studies in Family Planning. 2014; 45(2): 123-150.
3. Sarnak D, Tsui A, Makumbi F, Kibira SPS, Ahmed S. The predictive utility of unmet need on time to contraceptive adoption: A panel study of non-contracepting Ugandan women. Contraception. 2020; 2. https://doi.org/10.1016/j.conx.2020.100022.
4. Sarnak DO, Tsui A, Makumbi F, Kibira SPS, Ahmed S. A panel study of fertility preferences and contraceptive dynamics in the presence of competing pregnancy risks in Uganda. Demography. 2021; 58(1): 295-320. doi:10.1215/00703370-8937285.
5. Speizer IS, Calhoun L, Hoke T, Sengupta R. Measurement of unmet need for family planning: Longitudinal analysis of the impact of fertility desires on subsequent childbearing behaviors among urban women from Uttar Pradesh India. Contraception. 2013; 88(4):553–560. http://dx.doi.org.libproxy.lib.unc.edu/10.1016/j.contraception.2013.04.006.
6. Roy TK, Ram F, Nangla P, Saha U, Khan N. Can women's childbearing and contraceptive intentions predict contraceptive demand? Findings from a longitudinal study in central India. International Family Planning Perspectives. 2003; 29(1): 25-31.
7. Moreau C, Shankar M, Helleringer S, Becker S. Measuring unmet need for contraception as a point prevalence. BMJ Global Health 2019;4:e001581. doi:10.1136/bmjgh-2019-001581.
8. Panda BK, Ram F, Singh G, Saggurti N. Intention to use contraception among women in unmet need and not in unmet need in India. Demography India 2020; 41(2): 22-34.

For peer review only - http://bmjopen.bmj.com/site/about/guidelines.xhtml

9. Chandra-Mouli V, McCarraher DR, Phillips SJ, Williamson NE, Hainsworth G. Contraception for adolescents in low and middle income countries: Needs, barriers, and access. Reproductive Health. 2014; 11(1). https://doi.org/10.1186/1742-4755-11-1.

10. Ahinkorah BO. Predictors of unmet need for contraception among adolescent girls and young women in selected high fertility countries in sub-Saharan Africa: A multilevel mixed effects analysis. PLoS ONE. 2020; 15(8): e0236352. https://doi.org/10.1371/journal.pone.0236352.

11. Behrman JA, Wright KQ, Grant MJ, Soler-Hampejsek E. Trends in modern contraceptive use among young adult women in sub-Saharan Africa 1990 to 2014. Studies in Family Planning. 2018; 49(4):319–44. https://doi.org/10.1111/sifp.12075 PMID: 30431643.

12. Makola L, Mlangeni L, Mabaso M, Chibi B, Sokhela Z, Silimfe Z, et al. Predictors of contraceptive use among adolescent girls and young women (AGYW) aged 15 to 24 years in South Africa: results from the 2012 national population-based household survey. BMC Women's Health. 2019; 19(1):158. https://doi.org/10.1186/s12905-019-0861-8 PMID: 31830982.

13. Sidze EM, Lardoux S, Speizer IS, Faye CM, Mutua MM, Badji F. Young women access and use of contraception: The role of providers' restrictions in urban Senegal. International Perspectives on Sexual and Reproductive Health. 2014; 40(4):176. https://doi.org/10.1363/4017614 PMID: 25565345.

14. International Institute for Population Sciences (IIPS) and ICF. 2021. National Family Health Survey (NFHS)-5, State and District Factsheets, Uttar Pradesh. Mumbai: IIPS.

15. International Institute for Population Sciences (IIPS) and ICF. 2020. National Family Health Survey (NFHS)-5, State and District Factsheets, Bihar. Mumbai: IIPS.

16. International Institute for Population Sciences (IIPS) and ICF. 2021. National Family Health Survey (NFHS-5), India 2019-2020. Mumbai: IIPS.

For peer review only - http://bmjopen.bmj.com/site/about/guidelines.xhtml

For peer review only

[dataset] 17. Population Council, 2017, "UDAYA, Adolescent Survey, Bihar and Uttar Pradesh, 2015-16", https://doi.org/10.7910/DVN/RRXQNT, Harvard Dataverse, V2.

18. Santhya, K. G., R. Acharya, N. Pandey et al. 2017. Understanding the lives of adolescents and young adults (UDAYA) in Uttar Pradesh, India. New Delhi: Population Council.

19. Santhya, K. G., R. Acharya, N. Pandey et al. 2017. Understanding the lives of adolescents and young adults (UDAYA) in Bihar, India. New Delhi: Population Council.

[dataset] 20. Santhya, KG, 2020, "UDAYA, Adolescent Survey, Bihar and Uttar Pradesh, 2018–19", https://doi.org/10.7910/DVN/ZJPKW5, Harvard Dataverse, V2.

21. Koenig MA, Acharya K, Singh S, Roy TK. Do current measurement approaches underestimate levels of unwanted childbearing? Evidence from rural India. Population Studies. 2006;60(3):243–56.

22. International Institute for Population Sciences (IIPS) and ICF. 2017a. National Family Health Survey (NFHS-4), India, 2015-16: Uttar Pradesh. Mumbai: IIPS.

23. International Institute for Population Sciences (IIPS) and ICF. 2017b. National Family Health Survey (NFHS-4), India, 2015-16: Bihar. Mumbai: IIPS.

24. Senderowicz L, Maloney N. Suppl-side versus demand-side unmet need: Implications for family planning programs. Population and Development Review 2022; https://doi.org/10.1111/padr.12478.

25. Speizer IS, Bremner J, Farid S, et al. Improving family planning indicators of unmet need and demand satisfied. Global Health Science and Practice 2022;10(1): e2100450; https://doi.org/10.9745/GHSP-D-21-00450.

26. Pandey A, Singh KK. Contraceptive use before first pregnancy by women in India (2005–2006): determinants and differentials. BMC Public Health. 2015; 15, 1316. https://doi.org/10.1186/s12889-015-2652-y.

For peer review only - http://bmjopen.bmj.com/site/about/guidelines.xhtml

27. Coll CVN, Ewerling F, Hellwig F, de Barros AJD. Contraception in adolescence: The influence of parity and marital status on contraceptive use in 73 low-and middle-income countries. Reproductive Health. 2019; 16, 21. https://doi.org/10.1186/s12978-019-0686-9.

28. Achyut P, Mishra A, Montana L, Sengupta R, Calhoun LM, Nanda P. Integration of family planning with maternal health services: An opportunity to increase postpartum modern contraceptive use in urban Uttar Pradesh, India. Journal of Family Planning and Reproductive Health Care. 2016;42:107-115.

For peer review only - http://bmjopen.bmj.com/site/about/guidelines.xhtml

Declarations

Author affiliations:
[1]Carolina Population Center, University of North Carolina at Chapel Hill, Chapel Hill, North Carolina, USA
[2]Department of Maternal and Child Health, Gillings School of Global Public Health, University of North Carolina at Chapel Hill, Chapel Hill, North Carolina, USA
[3]Population Council, New Delhi, India
[4]Bill & Melinda Gates Foundation, Seattle, Washington and New Delhi, India

Contributors: ISS conceptualized the study and performed data analyses; AJFZ provided analysis inputs, including coding of some key variables; AJFZ, LMC, PN, NS and GW provided insights into the study design and analysis approach. All authors reviewed drafts of the paper and approved the final version of the paper.

Funding: This research uses data from the study on "Understanding the Lives of Adolescents and Young Adults (UDAYA) in Bihar and Uttar Pradesh" which was collected by the Population Council. Data collection was funded by Bill & Melinda Gates Foundation (OPP1111281) and the David and Lucile Packard Foundation (# 2014-40467). This work was also supported by the Bill & Melinda Gates Foundation (INV-009814). Under the grant conditions of the Foundation, a Creative Commons Attribution 4.0 Generic License has already been assigned to the Author Accepted Manuscript version that might arise from this submission. We also received general support from the Population Research Infrastructure Program through an award to the Carolina Population Center (P2C HD050924) at The University of North Carolina at Chapel Hill.

Competing interests: None declared

Patient consent for publication: Not required

Ethics approval: For the primary data collection, ethics approval was received by the Institutional Review Board at the Population Council (protocol number is 698). All individuals provided consent (written was optional) to participate. This study uses secondary de-identified data and was deemed exempt from ethical review by the University of North Carolina Institutional Review Board (protocol number 21-2643).

Data availability: All data used for this paper are publicly available on Harvard Dataverse at: https://doi.org/10.7910/DVN/RRXQNT (Wave 1 data); https://doi.org/10.7910/DVN/ZJPKW5 (Wave 2 data).

For peer review only - http://bmjopen.bmj.com/site/about/guidelines.xhtml

STROBE Statement—checklist of items that should be included in reports of observational studies

	Item No	Recommendation	Page No
**Title and abstract**	1	(*a*) Indicate the study's design with a commonly used term in the title or the abstract	1
		(*b*) Provide in the abstract an informative and balanced summary of what was done and what was found	1
**Introduction**			
Background/rationale	2	Explain the scientific background and rationale for the investigation being reported	3-4
Objectives	3	State specific objectives, including any prespecified hypotheses	5
**Methods**			
Study design	4	Present key elements of study design early in the paper	5
Setting	5	Describe the setting, locations, and relevant dates, including periods of recruitment, exposure, follow-up, and data collection	5
Participants	6	(*a*) *Cohort study*—Give the eligibility criteria, and the sources and methods of selection of participants. Describe methods of follow-up<br>*Case-control study*—Give the eligibility criteria, and the sources and methods of case ascertainment and control selection. Give the rationale for the choice of cases and controls<br>*Cross-sectional study*—Give the eligibility criteria, and the sources and methods of selection of participants	5
		(*b*) *Cohort study*—For matched studies, give matching criteria and number of exposed and unexposed<br>*Case-control study*—For matched studies, give matching criteria and the number of controls per case	5
Variables	7	Clearly define all outcomes, exposures, predictors, potential confounders, and effect modifiers. Give diagnostic criteria, if applicable	6-10
Data sources/ measurement	8*	For each variable of interest, give sources of data and details of methods of assessment (measurement). Describe comparability of assessment methods if there is more than one group	*6-10*
Bias	9	Describe any efforts to address potential sources of bias	6
Study size	10	Explain how the study size was arrived at	NA (secondary data)
Quantitative variables	11	Explain how quantitative variables were handled in the analyses. If applicable, describe which groupings were chosen and why	6-10
Statistical methods	12	(*a*) Describe all statistical methods, including those used to control for confounding	12
		(*b*) Describe any methods used to examine subgroups and interactions	
		(*c*) Explain how missing data were addressed	8
		(*d*) *Cohort study*—If applicable, explain how loss to follow-up was addressed	

For peer review only

Case-control study—If applicable, explain how matching of cases and controls was addressed<br>*Cross-sectional study*—If applicable, describe analytical methods taking account of sampling strategy	
(*e*) Describe any sensitivity analyses	

Continued on next page

For peer review only

For peer review only - http://bmjopen.bmj.com/site/about/guidelines.xhtml

Results			
Participants	13*	(a) Report numbers of individuals at each stage of study—eg numbers potentially eligible, examined for eligibility, confirmed eligible, included in the study, completing follow-up, and analysed	13, Table 1
		(b) Give reasons for non-participation at each stage	
		(c) Consider use of a flow diagram	
Descriptive data	14*	(a) Give characteristics of study participants (eg demographic, clinical, social) and information on exposures and potential confounders	Table 1
		(b) Indicate number of participants with missing data for each variable of interest	
		(c) *Cohort study*—Summarise follow-up time (eg, average and total amount)	
Outcome data	15*	*Cohort study*—Report numbers of outcome events or summary measures over time	*13-18; Table 1*
		Case-control study—Report numbers in each exposure category, or summary measures of exposure	
		Cross-sectional study—Report numbers of outcome events or summary measures	
Main results	16	(*a*) Give unadjusted estimates and, if applicable, confounder-adjusted estimates and their precision (eg, 95% confidence interval). Make clear which confounders were adjusted for and why they were included	13-18; Tables 2-4
		(*b*) Report category boundaries when continuous variables were categorized	
		(*c*) If relevant, consider translating estimates of relative risk into absolute risk for a meaningful time period	
Other analyses	17	Report other analyses done—eg analyses of subgroups and interactions, and sensitivity analyses	13-18; Table 5
**Discussion**			
Key results	18	Summarise key results with reference to study objectives	18-19
Limitations	19	Discuss limitations of the study, taking into account sources of potential bias or imprecision. Discuss both direction and magnitude of any potential bias	20-21
Interpretation	20	Give a cautious overall interpretation of results considering objectives, limitations, multiplicity of analyses, results from similar studies, and other relevant evidence	18-19
Generalisability	21	Discuss the generalisability (external validity) of the study results	18-19
**Other information**			
Funding	22	Give the source of funding and the role of the funders for the present study and, if applicable, for the original study on which the present article is based	27

*Give information separately for cases and controls in case-control studies and, if applicable, for exposed and unexposed groups in cohort and cross-sectional studies.

Note: An Explanation and Elaboration article discusses each checklist item and gives methodological background and published examples of transparent reporting. The STROBE checklist is best used in conjunction with this article (freely available on the Web sites of PLoS Medicine at http://www.plosmedicine.org/, Annals of Internal Medicine at http://www.annals.org/, and Epidemiology at http://www.epidem.com/). Information on the STROBE Initiative is available at www.strobe-statement.org.

For peer review only - http://bmjopen.bmj.com/site/about/guidelines.xhtml
